# Reactome pathway analysis from whole-blood transcriptome reveals unique characteristics of systemic sclerosis patients at the preclinical stage

**DOI:** 10.3389/fimmu.2023.1266391

**Published:** 2023-11-03

**Authors:** Chiara Bellocchi, Xuan Wang, Marka A. Lyons, Maurizio Marchini, Maurizio Lorini, Vincenzo Carbonelli, Nicola Montano, Shervin Assassi, Lorenzo Beretta

**Affiliations:** ^1^ Department of Clinical Sciences and Community Health, University of Milan, Milan, Italy; ^2^ Scleroderma Unit, Referral Center for Systemic Autoimmune Diseases, Fondazione IRCCS Ca’ Granda, Ospedale Maggiore Policlinico, Milan, Italy; ^3^ Biostatistics, Baylor Institute for Immunology Research, Dallas, TX, United States; ^4^ Division of Rheumatology, The University of Texas Health Science Center at Houston, Houston, TX, United States

**Keywords:** systemic sclerosis, preclinical systemic sclerosis, pathways, disease progression, gene expression

## Abstract

**Objective:**

This study aims to characterize differential expressed pathways (DEP) in subjects with preclinical systemic sclerosis (PreSSc) characterized uniquely by Raynaud phenomenon, specific autoantibodies, and/or capillaroscopy positive for scleroderma pattern.

**Methods:**

Whole-blood samples from 33 PreSSc with clinical prospective data (baseline and after 4 years of follow-up) and 16 matched healthy controls (HC) were analyzed for global gene expression transcriptome analysis via RNA sequencing. Functional Analysis of Individual Microarray Expression method annotated Reactome individualized pathways. ANOVA analysis identified DEP whose predictive capability were tested in logistic regression models after extensive internal validation.

**Results:**

At 4 years, 42.4% subjects progressed (evolving PreSSc), while the others kept stable PreSSc clinical features (stable PreSSc). At baseline, out of 831 pathways, 541 DEP were significant at a false discovery rate <0.05, differentiating PreSSc *versus* HC with an AUROC = 0.792 ± 0.242 in regression models. Four clinical groups were identified via unsupervised clustering (HC, HC and PreSSc with HC-like features, PreSSc and HC with PreSSc-like features, and PreSSc). Biological signatures changed with disease progression while remaining unchanged in stable subjects. The magnitude of change was related to the baseline cluster, yet no DEP at baseline was predictive of progression. Disease progression was mostly related to changes in signal transduction pathways especially linked to calcium-related events and inositol 1,4,5-triphosphate metabolism.

**Conclusion:**

PreSSc had distinguished Reactome pathway signatures compared to HC. Progression to definite SSc was characterized by a shift in biological fingertips. Calcium-related events promoting endothelial damage and vasculopathy may be relevant to disease progression.

## 1 Introduction

Systemic sclerosis (SSc) is a complex autoimmune disease characterized by immune system activation, widespread vasculopathy, collagen deposition, and extracellular matrix deposition, eventually leading to organ fibrosis (1). SSc usually develops as a progressive disease, and a preclinical phase (PreSSc) has been recognized where the prototypical clinical alterations of fibrotic SSc cannot yet be observed (2). Indeed mounting evidences suggest that the very early SSc phases (either labeled as early SSc, (EaSSc) (3), undifferentiated connective tissue disease at risk for SSc, UCTD-SSc (4), very early diagnosis of SSc (VEDOSS) (5), or PreSSc) have a cumulative risk of developing a definite SSc equal to 50% within 5 years from the diagnosis (5–7). According to the LeRoy and Medsger criteria, PreSSc are characterized by the combination of Raynaud’s phenomenon (RP) along with either prototypical nailfold capillaroscopy (NVC) alterations and/or presence of SSc-related autoantibodies (3). Recently, we demonstrated that, biologically, PreSSc already show alterations in circulating biomarkers along with functional immune system alterations that distinguish them from healthy controls (HCs) or definite subsets of SSc (7).

Transcriptome sequencing, also called RNA sequencing (RNAseq), is an omic technique that allows the deep evaluation of the transcriptional status of biological samples to assess which genes are active under specific conditions. The evaluation of differentially expressed (DE) genes is usually the first step in RNAseq analysis, yet it often lacks adequate power or immediate interpretability. It has been extensively demonstrated that combining genes in a set of interest as, for instance, those converging to certain biological pathways may circumvent several problems related to single-gene analysis (8). The collection of gene sets allows to analyze the joint activity of biologically related transcripts and provides the groundwork for an easy interpretation of results. Among the several existing methods, the Functional Analysis of Individual Microarray (or RNA-seq) Expression (FAIME) algorithm (9) proved both powerful and capable of controlling type I errors, providing unsupervised individual measurements of mechanisms that are not based on sample-wise statistics and are thus useful for individualized predictions or comparisons. Its applicability in SSc patients has recently been demonstrated in a large European project, allowing the discovery of several deregulated pathways relevant to SSc pathogenesis (10).

In the present work, we aim at analyzing DE pathways (DEP) obtained from whole-blood transcripts of PreSSc through the FAIME algorithm to characterize the biological alterations that may be relevant to the early development of SSc and progression.

## 2 Materials and methods

### 2.1 Patients

Thirty-three PreSSc according to 2001 LeRoy and Medsger criteria (3) and previously described elsewhere (7) were included. All the subjects underwent a complete clinical evaluation for 4 years to detect the occurrence of additional features indicative of definite SSc progression according to the 2013 ACR/EULAR classification criteria (2, 7). Video-capillaroscopy was performed only at baseline for diagnostic purposes to assess the presence of secondary Raynaud’s phenomenon in PreSSc. Patients progressing toward definite SSc were labeled as “evolving PreSSc”, while the others were labeled as “stable PreSSc”. None of the subjects were treated with immunosuppressants or immunomodulators both at baseline and at follow-up. The subjects were only treated with low-dose aspirin and/or calcium channel blockers for the control of RP. Blood samples from patients along with samples from 16 age-, gender-, and ethnically matched and unrelated healthy controls (HC) were collected in RNA stabilizers (PAX gene tubes®) and stored at -80° for later analysis; the patients’ samples were collected both at baseline and at the end of observation (4th year). The data and sample collection was approved by the local Ethical Committee Comitato Etico Milano Area 2 (approval no. 559_2018); all subjects provided signed informed consent.

### 2.2 RNA sequencing

RNA was extracted according to the manufacturer’s protocol, and quality was assessed by using Agilent 2100 Bioanalyzer (Agilent Technologies). Globin genes were depleted using GLOBIN Clear kit. mRNA was enriched from total RNA using oligo(dt) beads (NEB Next Ultra II RNA Kit following the poly(A) enrichment workflow). The mRNA was fragmented randomly in fragmentation buffer and reverse-transcribed to cDNA. The cDNAs were converted to double-stranded cDNAs and then subjected to end-repair, A-tailing, adapter ligation, size selection, and PCR enrichment. The library concentration was first quantified using a Qubit 2.0 fluorometer (Life Technologies) and then diluted to 1 ng/uL before checking the insert size on Agilent 2100 and quantifying to greater accuracy by quantitative PCR (q-PCR) (library activity, >2 nM). The libraries were pooled into Novaseq6000 machines according to molarity and expected data volume. A paired-end 150-bp sequencing strategy was used to generate an average of 88 million reads per sample. Transcript data were normalized with DESeq2. The transcripts were filtered with count-per-million (CPM), keeping those genes which have at least two samples with CPM >1. A total of 17,746 transcripts out of 60,641 in raw count file passed the filtering criteria.

### 2.3 Functional annotation and differential expression pathway analysis

Individual functional annotations were performed with the FAIME algorithm (9) considering Reactome pathways mapped by at least five genes/transcripts (https://reactome.org); Entrez IDs without official gene symbols were dropped from the analysis. The FAIME scores were normalized in the [-1, 1] closed interval for visualization by heat maps. Data were aggregated via unsupervised clustering (hierarchical method via Ward’s linkage) and manually annotated.

Because the FAIME scores are a composite measure of transcripts, fold change cannot be used to measure the difference in the expression level of pathways, and thus effect size was used instead. Winsorized data were used to calculate the robust effect size (dR) and to perform the *t*-test analysis. Annotations with a false discovery rate (FDR) *p* < 0.05 and with a |dR | > 0.62897, corresponding to a moderate effect size (11), were considered significant.

The predictive capability of DEP was tested in logistic regression models with L2 penalty after 20 runs of 10-fold cross-validation, where pathways of interest were selected in training sets by applying the abovementioned criteria, and the results from independent test sets were averaged.

Changes in FAIME Reactome scores between baseline and end of observation were determined via the ANCOVA change method (difference between post- and pre- values, correcting for baseline).

The capability of baseline Reactome scores to predict evolution was modeled via machine learning algorithms (logistic regression, naïve Bayes classifiers, or random forest) via 20 runs of 10-fold cross-validation with univariate selection of relevant features (either 1%, 5%, 10%, or 20% of the total) in each training set via nested cross-validation.

For all the analyses, custom codes written in python by LB on top of the Scikit-learn library (https://scikit-learn.org/stable/index.html) were used.

## 3 Results

The clinical and demographic characteristics are summarized in **Table 1**. Overall, 14 (42.4%) patients had signs of progression at 4 years; the autoantibody profile, demographic and clinical features such as Raynaud phenomenon duration, forced vital capacity, and diffusing capacity for carbon monoxide were comparable at baseline among stable and evolving PreSSc, and no variables were associated with progression toward a definite SSc. Only the proportion of subjects taking low-dose aspirin at baseline was significantly higher in stable PreSSc (**Table 1**). In **Supplementary Tables S1**, **S2**, the clinical features of stable and evolving PreSSc are shown and detailed for each patient. Specifically, stable PreSSc did not develop skin fibrosis, while 12 evolving PreSSc presented limited cutaneous SSc (lcSSc) features, mostly puffy fingers (eight cases).

**Table 1:** Baseline clinical characteristics of PreSSc with stratification according to the 4-year progression outcome.

Features	All SSc *n* = 33	Stable PreSSc *n* = 19	Evolving PreSSc *n* = 14	HC *n* = 16
Age, mean years ± SD	56.6 ± 12.6	57.1 ± 10.4	55.9 ± 15.4	55.3 ± 12.6
Gender, female, *n* (%)	28 (84.8)	15 (78.9)	13 (92.9)	15 (93.8)
Ethnicity, Caucasian, *n* (%)	33 (100)	19 (100)	14 (100)	16 (100)
ANA, *n* (%)	32 (97)	18 (94.7)	14 (100)	NA
ACA, *n* (%)	22 (66.7)	12 (63.2)	10 (71.4)	NA
Anti-Scl70, *n* (%)	6 (18.2)	3 (15.7)	3 (21.4)	NA
Others, *n* (%)	5 (15.1)	4 (21.1)	1 (7.1)	NA
RP years duration, mean ± SD	9.6 ± 8.1	9.9 ± 8.5	9.1 ± 7.8	NA
FVC (%), mean ± SD	115.5 ± 16.7	114.1 ± 18.3	117.4 ± 14.7	NA
DLCO (%), mean ± SD	86 ± 18.6	86.3 (20.3)	85.6 (16.8)	NA
SSc clinical features				
None, *n* (%)	16 (48.5)	10 (52.6)	6 (42.9)	NA
Skin, *n* (%)	0	0	0	NA
Upper GI, *n* (%)	10 (30.3)	4 (21.1)	4 (28.6)	NA
Teleangectasia, *n* (%)	0	0	0	NA
Low-dose aspirin, *n* (%)	28 (84.5)	19 (100)^a^	9 (64.3)	NA
CCB, *n* (%)	24 (72.7)	15 (78.9)	8 (57.1)	NA

a p value = 0.008 based on chi-square test.

ANA, anti-nuclear antibodies; ACA, anti-centromere antibodies; RP, Raynaud phenomenon; FVC, forced vital capacity; DLCO, diffusing capacity of the lung for carbon monoxide; N, number; GI, gastro-intestinal; CCB, calcium channel blockers; NA, not applicable; SD, standard deviation.

### 3.1 Baseline Reactome pathways

**Figure 1:**
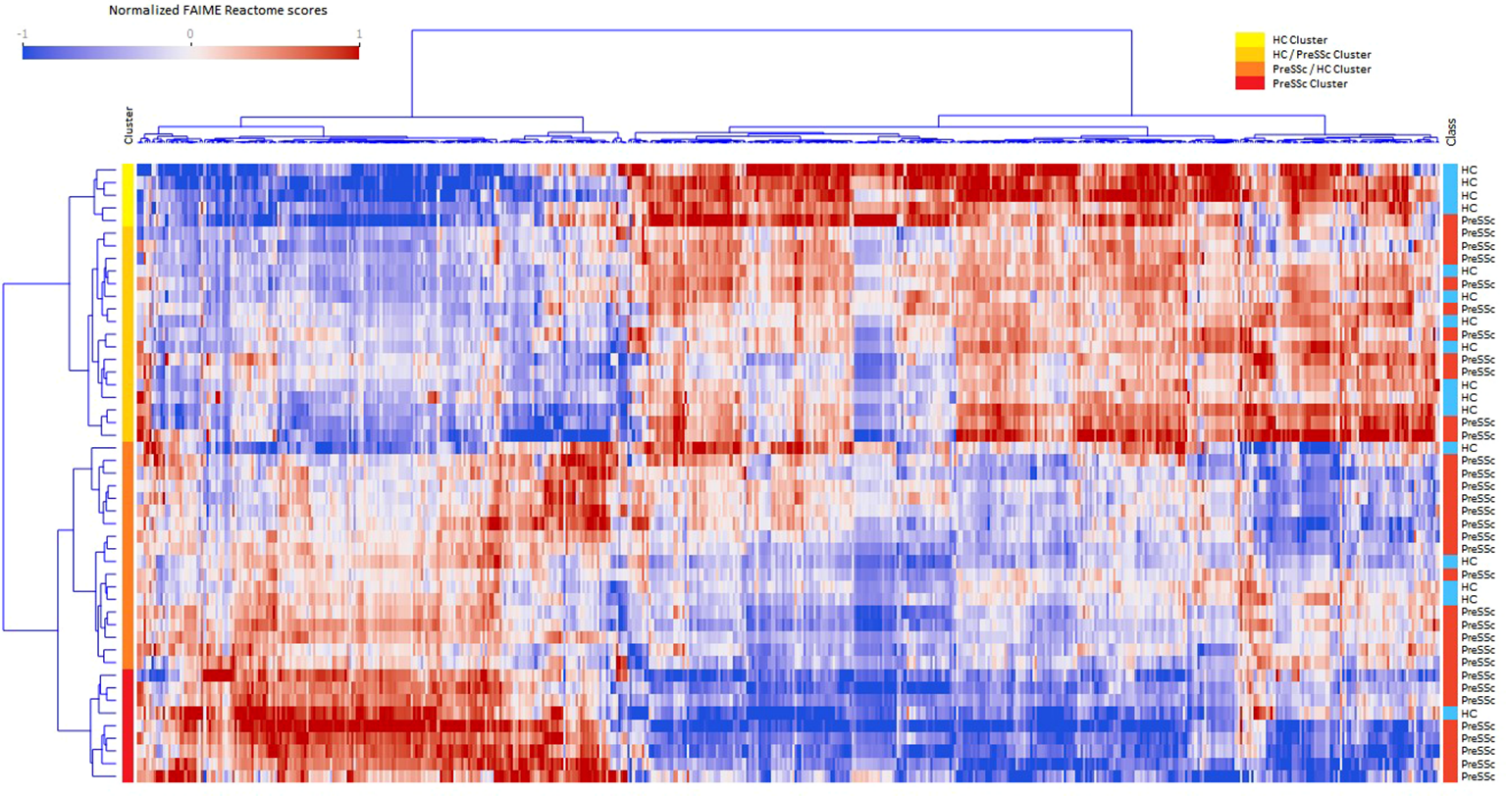
Heat maps of differentially expressed Reactome pathways at baseline. Heat maps after unsupervised clustering analysis of the 541 Reactome pathways that were differentially expressed in preclinical systemic sclerosis patients (PreSSc) vs. healthy controls (HCs) (false discovery rate < 0.05 and moderate effect size) at baseline. The subjects can be grouped in four clusters (from top to bottom, yellow to red bars) that are enriched in HCs (top) or PreSSc (bottom) and with a clearly visible gradient of expression profile.

A total of 831 Reactome pathways were explored; of these, 541 were significant at a FDR <0.05 and with a moderate effect size for the comparison PreSSc vs. HC; these DEPs are represented in the heat map in **Figure 1** and are detailed in the **Supplementary Material**; **Sheet 1**. According to the Reactome pathways, at least four clinical clusters could be identified after unsupervised analysis and annotation (from top to bottom), characterizing HC, HC, and PreSSc with HC-like features, PreSSc and HC with PreSSc-like features, and PreSSc. No clinical or laboratory characteristic was statistically associated with any of the PreSSc clusters (chi-square test or ANOVA was not significant for any variable).

To provide a better interpretation of DEPs and clusters, Reactome pathways were collapsed to their root location in the pathway browser as illustrated in **Supplementary Figure S1**. Overall, 18 pathways were represented (cell cycle, cellular response to stimuli, DNA repair, developmental biology, disease, extracellular matrix organization, gene expression–transcription, hemostasis, immune system, metabolism, metabolism of RNA, metabolism of proteins, neuronal system, organelle biogenesis and maintenance, signal transduction, transport of small molecules, vesicle-mediated transport); for the vast majority of them a clear-cut increasing or decreasing expression gradient across clinical clusters could be observed. An illustrative example is, for instance, that of DEPs related to the extracellular matrix organization (**Supplementary Figure S1**) that are more expressed at baseline in the PreSSc- and PreSSc/HC-enriched clusters than in the HC and HC/PreSSc (yellow and orange bar) clusters. Similar observations can be made for DEPs belonging to the immune system, where complement activation pathways are more expressed in the PreSSc- and PreSSc/HC-enriched clusters (**Supplementary Figure S1**) than in the HC and the HC/PreSSc clusters.

Overall, DEPs could jointly well differentiate PreSSc and HC with an AUROC = 0.792 ± 0.242 after 20 runs of internal 10-fold cross-validation with L2 penalized logistic regression models (**Supplementary Figure S2**). No functional pathway at baseline was found to be predictive of progression after evaluation via machine learning models (AUROC < 0.6).

### 3.2 Follow-up vs. baseline Reactome pathways

**Figure 2:**
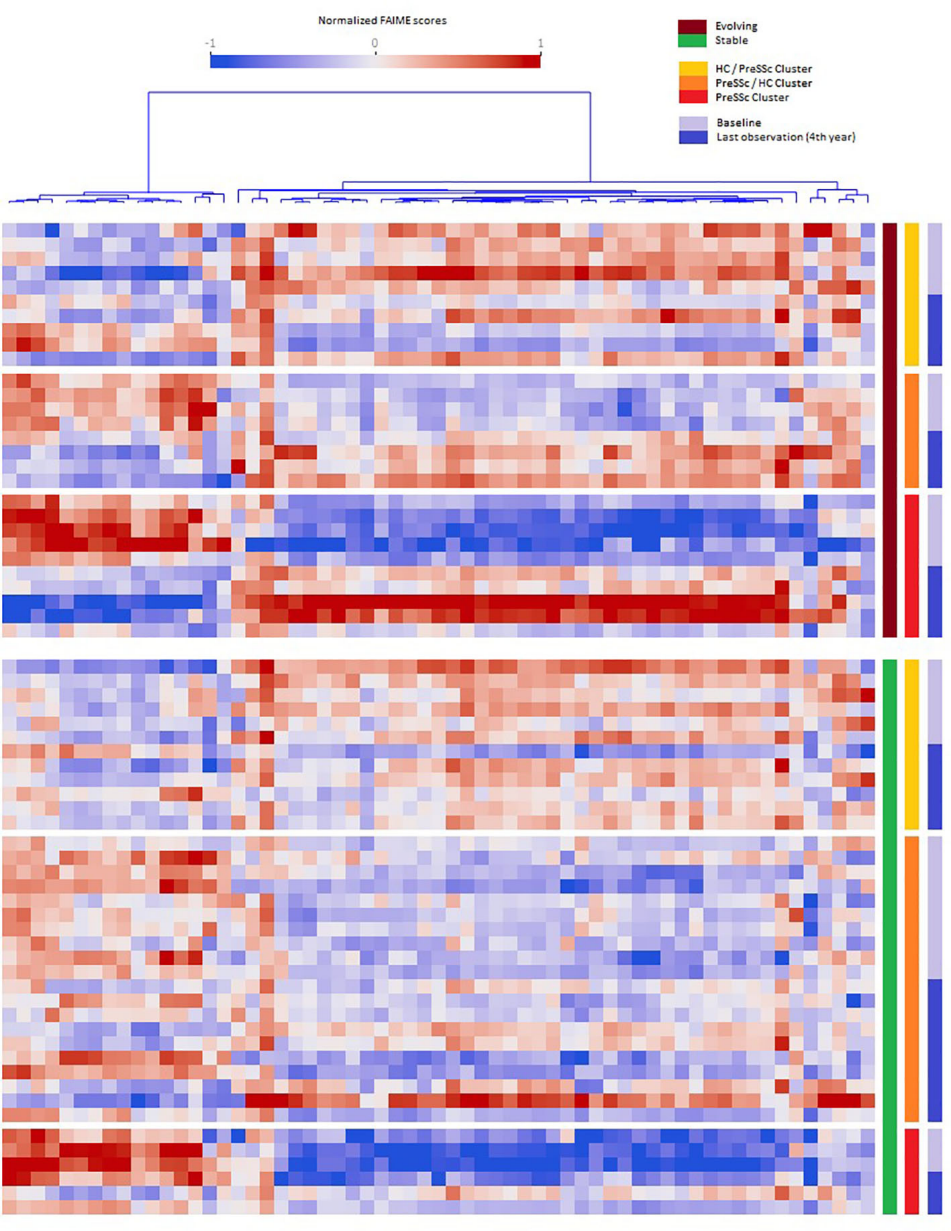
Baseline vs. follow-up differential expression pathway analysis. Heat maps representing the pathways whose expression significantly changed in the pre- and post-analysis in evolving vs. stable preclinical systemic sclerosis (PreSSc) and according to the baseline cluster (see **Figure 1**). In evolving patients (top charts, brown bar), there is a clear shift in the expression profile, especially in the PreSSc-enriched cluster(s); in stable patients, the expression profile remains unaltered.

When analyzing the post–pre change in Reactome pathways in ANCOVA models that included baseline expression profiles and clinical clusters, several pathways were found to be significantly associated with the future status of patients (progression vs. no progression). **Figure 2** shows that evolving PreSSc (brown bar) changed their biological signature, especially in the PreSSc cluster (red bar) and PreSSc/HC-enriched cluster (orange bar), while the Reactome pathway scores remained unchanged in stable patients (green bar).

The Reactome pathways related to disease progression are detailed in **Supplementary Material**; **Sheet 2**. The proportion of signal transduction pathways significantly associated with disease progression exceeded that of those without a significant association (*p* < 0.05, chi-square test). Specifically, a number of pathways related to inositol 1,4,5-triphosphate (IP3) signaling, calcium-dependent mechanisms, and related metabolic functions were found among those positively associated with progression. Interestingly, two pathways were related specifically to the immune system biological function: the interleukin 37 (IL-37) signaling and the Fc gamma receptor. We observed that the IL-37 signaling scores decreased in evolving PreSSc (pre: 1.286, post: -0.904), while they remained increased in stable subjects (pre: 2.502, post: 2.766). Other pathways of interest with positive association included the semaphorins pathway.

## 4 Discussion

PreSSc are SSc subjects at a preclinical stage whose unique clinical feature is the occurrence of RP and do not present any additional fibrotic feature or involvement of internal organs. As already described in a previous study of ours, the whole-blood transcriptome profile of these patients is different as compared to HCs (7). Accordingly, we here confirm that 541 Reactome pathways, belonging to 18 functional groups (among them cell cycle, cellular response to stimuli, DNA repair, extracellular matrix organization, gene expression–transcription, and immune system) clearly distinguished the profile of PreSSc from HCs. After unsupervised clustering analysis, we observed that PreSSc progressively stratified from a cluster with HC-like features to a cluster enriched with PreSSc subjects and with clear-cut different biological characteristics (**Figure 1**). This observation reinforces the notion that, from the biological point of view, SSc is a heterogeneous disease, regardless of the clinical phenotype or main laboratory characteristics that indeed were not related to functional clusters. Many of baseline DEPs, as for instance those related to collagen synthesis and degradation, increased B cell activation, and complement activation (12), are associated to pathogenetic events already documented in definite SSc. Others, on the contrary, have a less straightforward interpretation, as for instance the downregulation of pathways related to interleukin signaling or cell cycle or signal transduction.

A remarkable finding of ours is that the signatures of PreSSc changed over time only in those subjects who did progress, while they remained unaltered in stable patients (**Figure 2**) even though a specific “signature of progression” cannot be sorted out. Indeed while in the vast majority of evolving patients a well-defined direction of change can be observed (i.e., from high to low relative expression in the pathways that cluster on the left in **Figure 2**), an exactly opposite phenomenon can be observed in others (i.e., from low to high relative expression). Interestingly, the degree of biological shift is the highest in cluster 3, that is, the one at baseline was most notably different from HCs (**Figure 1**). On the contrary, patients belonging to the HC-like cluster experienced a lesser degree of biological shift when progressing and, most noticeably, maintained a different signature as compared to clusters 2 and 3 patients. Interestingly, among the pathways, IL-37 signaling specifically related to the immune system biological function showed a negative association with progression. IL-37 is a cytokine that belongs to the IL-1 family with anti-inflammatory properties; its specific role in SSc has not been fully elucidated yet (13). Interestingly, the semaphorin pathway showed a positive association with progression. Semaphorins are receptors with a role in regulating angiogenesis and neurovascular alterations. From recent studies, semaphorins are associated with vascular manifestations in SSc, ranging from microvascular deregulation to digital ulcer occurrence (14). Overall, these findings seem to indicate that the clinical heterogeneity observed in preclinical subjects is maintained in patients with definite disease, not dissimilarly from the heterogeneity described elsewhere in fibrotic tissue samples (15). Taken together, our results suggest that a single therapeutic intervention is unlikely to tackle disease progression in all PreSSc subjects and that a careful biological stratification is desirable to intercept SSc from the preclinical stage—for instance, our results suggest that IP3-related pathways and the accumulation of intracellular calcium are a potential area of intervention, most likely because these pathways may enhance vasculopathy, capillary leaking, and contraction (16), in line with the notion that calcium channel blockers are the anchor therapy of Raynaud’s phenomenon (e.g., the cardinal feature of PreSSc) with several positive effects beyond vasodilation (17). However, some patients—namely, those in cluster 1—do have a reduced expression of these pathways despite progression, at variance with the majority of subjects, as those belonging to clusters 2 and 3.

While our study presents several strengths, including the careful characterization of PreSSc cohort as well as the longitudinal collection of samples, we shall acknowledge some limitations. These include the relatively small sample size due to the difficulties in intercepting SSc patients at a preclinical stage. Moreover, we experienced difficulties in building predictive models of disease progression that cannot thus be forecasted on the basis of clinical or biological characteristics. This is undoubtedly linked to the fact that many pathways do not change in non-progressing patients (**Figure 2**) and that this behavior is unrelated to individualized pathway expression.

In summary, we observed that differentially expressed pathways characterize subjects at a preclinical stage of SSc and that biomolecular changes are peculiar of PreSSc that over time clinically progressed into definite diseases. Furthers studies are worth to be conducted on PreSSc patient cohorts to detect and confirm the signatures that may herald possible therapeutic targets to prevent disease progression.

## Data availability statement

The data presented in the study are deposited in the Gene Expression Omnibus (GEO) repository, accession number GSE224849.

## Author contributions

CB: Conceptualization, Data curation, Formal Analysis, Funding acquisition, Methodology, Project administration, Visualization, Writing – original draft, Writing – review & editing. XW: Data curation, Formal Analysis, Investigation, Methodology, Software, Writing – review & editing. MAL: Data curation, Formal Analysis, Investigation, Methodology, Software, Writing – review & editing. MM: Data curation, Investigation, Methodology, Writing – review & editing. ML: Data curation, Investigation, Methodology, Writing – review & editing. VC: Data curation, Investigation, Methodology, Writing – review & editing. NM: Project administration, Supervision, Visualization, Writing – review & editing. SA: Conceptualization, Data curation, Formal Analysis, Funding acquisition, Investigation, Methodology, Project administration, Supervision, Visualization, Writing – review & editing. LB: Conceptualization, Data curation, Formal Analysis, Investigation, Methodology, Software, Supervision, Writing – original draft, Writing – review & editing.
