# Supplementary figures and images for: Reactome pathway analysis from whole-blood transcriptome reveals unique characteristics of systemic sclerosis patients at the preclinical stage
Source: Front Immunol. 2023 Nov 3;14:1266391. doi: 10.3389/fimmu.2023.1266391 (PMC10654742; doi:10.3389/fimmu.2023.1266391)

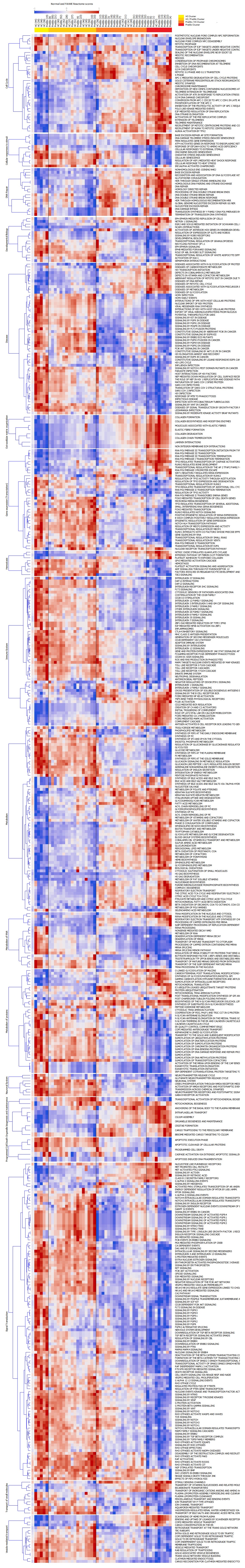

Supplement: Supplementary file 1 [file Image_1.jpeg]
