# Supplementary material for: Reactome pathway analysis from whole-blood transcriptome reveals unique characteristics of systemic sclerosis patients at the preclinical stage
Source: Front Immunol. 2023 Nov 3;14:1266391. doi: 10.3389/fimmu.2023.1266391 (PMC10654742; doi:10.3389/fimmu.2023.1266391)
Supplement: Supplementary file 2 [file Table_2.docx]

**Supplementary Table 2: Demographic and clinical characteristics of individual patients**

| **ID** | **Gender** | **Age_BL** | **Time BL_FU** | **Auto AB** | **Cap_BL** | **Subset_BL** | **SSc_Feat_BL** | **Time RP_ BL** | **1st non RP Sign** | **Subset_FU** | **SSc_Feat_FU** | **Time RP at FU** | **Skin evaluation_FU** |
| --- | --- | --- | --- | --- | --- | --- | --- | --- | --- | --- | --- | --- | --- |
| 2 | F | 51 | 4,1 | ACA | Pos | PreSSc | E | 15,1 | E | PreSSc | E | 19,2 | Normal skin |
| 7 | M | 54 | 3,3 | ACA | Neg | PreSSc | None | 3,7 | None | PreSSc | None | 7,1 | Normal skin |
| 8 | F | 56 | 4,0 | ANA Nu | Pos | PreSSc | E | 1,1 | PF | PreSSc | None | 5,1 | Slight edema 2nd and 3rd finger dominant hand |
| 9 | F | 51 | 4,1 | ACA | Pos | PreSSc | DL | 2,1 | None | PreSSc | DL | 6,2 | Normal skin |
| 10 | F | 40 | 2,7 | ACA | Pos | PreSSc | DL | 0,6 | None | PreSSc | DL | 3,3 | Normal skin |
| 11 | F | 56 | 4,3 | Topo I | Pos | PreSSc | None | 2,6 | E | PreSSc | T (one) | 6,9 | Normal skin + only one telangiectasia |
| 12 | M | 67 | 3,2 | ACA | Pos | PreSSc | E | 14,1 | E | PreSSc | E | 17,3 | Normal skin |
| 16 | F | 60 | 4,5 | ACA | Pos | PreSSc | DL | 8,7 | E | PreSSc | DL | 13,3 | Normal skin |
| 20 | F | 80 | 4,8 | ACA | Pos | PreSSc | None | 9,5 | E | PreSSc | None | 14,3 | Normal skin |
| 21 | F | 72 | 4,4 | ANA Nu | Pos | PreSSc | None | 3,3 | None | PreSSc | None | 7,7 | Normal skin |
| 22 | F | 71 | 3,4 | ACA | Pos | PreSSc | E DL | 5,3 | E | PreSSc | E DL | 8,6 | Normal skin |
| 25 | F | 54 | 4,5 | Neg | Pos | PreSSc | E | 19,0 | E | PreSSc | E | 23,5 | Normal skin |
| 27 | F | 49 | 4,4 | ACA | Pos | PreSSc | E | 34,7 | None | PreSSc | E | 39,1 | Normal skin |
| 29 | F | 58 | 3,4 | ACA | Pos | PreSSc | None | 15,0 | U | PreSSc | None | 18,4 | Normal skin |
| 30 | F | 68 | 3,5 | ACA | Neg | PreSSc | None | 16,8 | E | PreSSc | E T (one) | 20,3 | One telangectasia 4th finger |
| 31 | M | 44 | 3,5 | Topo I | Pos | PreSSc | None | 9,9 | E | PreSSc | None | 13,4 | Normal skin |
| 32 | F | 53 | 3,5 | ANA Nu | Pos | PreSSc | None | 5,5 | None | PreSSc | None | 8,9 | Normal skin |
| 35 | F | 55 | 4,4 | ACA | Pos | PreSSc | None | 5,0 | Sicca | PreSSc | None | 9,4 | Normal skin |
| 52 | M | 46 | 4,7 | Topo I | Pos | PreSSc | None | 16,9 | E | PreSSc | None | 21,6 | Normal skin |
| 3 | F | 32 | 4,4 | Topo I | Pos | PreSSc | DL | 1,6 | ILD | lcSSc | PF, T | 6,0 | Puffy fingers, telangectasia |
| 5 | F | 83 | 4,0 | ACA | Pos | PreSSc | E DL | 23,2 | E | lcSSc | PF,T, sclerodactyly, Mouth Fibrosis | 27,2 | Puffy fingers, telangectasia |
| 33 | F | 68 | 4,0 | ACA | Pos | PreSSc | E | 21,5 | E | lcSSc | PF T E C sclerodactyly, Mouth Fibrosis | 25,6 | Puffy fingers, sclerodactyly right hand, mouth fibrosis |
| 34 | F | 49 | 2,9 | Topo I | Pos | PreSSc | None | 0,6 | PF | lcSSc | PF sclerodactyly II finger right hand | 3,5 | Puffy fingers and sclerodactyly  2nd finger right hand |
| 1 | F | 49 | 4,5 | ACA | Pos | PreSSc | None | 1,8 | PF | lcSSc | PF | 6,2 | Puffy fingers |
| 4 | F | 59 | 3,3 | ACA | Pos | PreSSc | DL | 13,1 | E | lcSSc | PF E DL | 16,4 | Puffy fingers |
| 13 | F | 70 | 4,4 | ACA | Pos | PreSSc | None | 1,0 | T | lcSSc | T | 5,4 | Two telangiectasia on the finger; |
| 15 | F | 54 | 4,7 | ACA | Pos | PreSSc | None | 17,1 | PF | lcSSc | PF E T(one) | 21,8 | Puffy fingers; one telangectasia |
| 18 | F | 27 | 4,8 | ACA | Pos | PreSSc | DL | 8,5 | E | lcSSc | DL E T | 13,3 | Telangectasia hands |
| 19 | M | 69 | 4,5 | ACA | Pos | PreSSc | E | 9,8 | E | lcSSc | E PF | 14,3 | Puffy fingers |
| 23 | F | 52 | 4,1 | ACA | Pos | PreSSc | None | 3,5 | E | lcSSc | PF T E | 7,6 | Puffy fingers, teleangectasia hands |
| 28 | F | 47 | 4,3 | Topo I | Pos | PreSSc | DL | 7,1 | E | lcSSc | DL E PF Mouth Fibrosis | 11,3 | Reduced mouth opening; puffy fingers |
| 54 | F | 53 | 4,9 | ANA Nu | Pos | PreSSc | None | 3,2 | E | lcSSc | PF T E | 8,1 | Puffy fingers, teleangectasia hands |
| 55 | F | 71 | 4,9 | ACA | Pos | PreSSc | E | 14,8 | E | lcSSc | PF E | 19,7 | Puffy fingers |

Abbreviations: BL: baseline; FU: follow-up; T: telangiectasia; PF: puffy fingers; E: Esophageal involvement; DL: DLCO reduction (<80%); ILD: Interstitial lung disease; C: calcinosis; ACA: anticentromere; ANA nu: antinuclear antibodies nucleolar; Topo 1: Anti topoisomerase (Scl70)
