# Supplementary material for: Reactome pathway analysis from whole-blood transcriptome reveals unique characteristics of systemic sclerosis patients at the preclinical stage
Source: Front Immunol. 2023 Nov 3;14:1266391. doi: 10.3389/fimmu.2023.1266391 (PMC10654742; doi:10.3389/fimmu.2023.1266391)
Supplement: Supplementary file 4 [file DataSheet_2.docx]

**Supplementary Table 1:** Subjects clinical features at follow-up

| **Features** | **Stable PreSSc**  **n = 19** | **Evolving PreSSc**  **n = 14** | **P value** |
| --- | --- | --- | --- |
| **RP years duration mean (SD)** | 13.9 (8.6) | 13.3 (7.8) | 0.849 |
| **FVC (%) mean (SD)** | 118.1 (18.1) | 118.8 (17.2) | 0.913 |
| **DLCO (%) mean (SD)** | 85.6 (23.2) | 77.2 (13.9) | 0.253 |
| **SSc clinical features** |  |  |  |
| **None n (%)** | 9 (47.4) | 0 | 0.004 |
| **lcSSc features n (%)** | 0 (0) | 12 (85.7) | <0.001 |
| **Puffy fingers n (%)** | 0 (0) | 8 (57.1) | <0.001 |
| **Sclerodactily n (%)** | 0 (0) | 4 (28.6) | 0.025 |
| **dcSSc n (%)** | 0 (0) | 0 (0) | 1 |
| **Upper GI n (%)** | 6 (31.6) | 9 (64.3) | 0.085 |
| **Teleangectasia n (%)** | 1 (5.3) | 5 (35.7) | 0.062 |
| **Low dose aspirin n (%)** | 14 (73.7) | 10 (71.4) | 1 |
| **CCB n (%)** | 13 (68.4) | 11 (78.6) | 0.698 |

Abbreviations: RP: Raynaud Phenomenon; FVC: forced vital capacity; DLCO: diffusing capacity of the lung for carbon monoxide; N: number; GI: gastro-intestinal; CCB: calcium channel blockers; SD: Standard deviation

**Supplementary** **Figure 2 –** Performance of classification


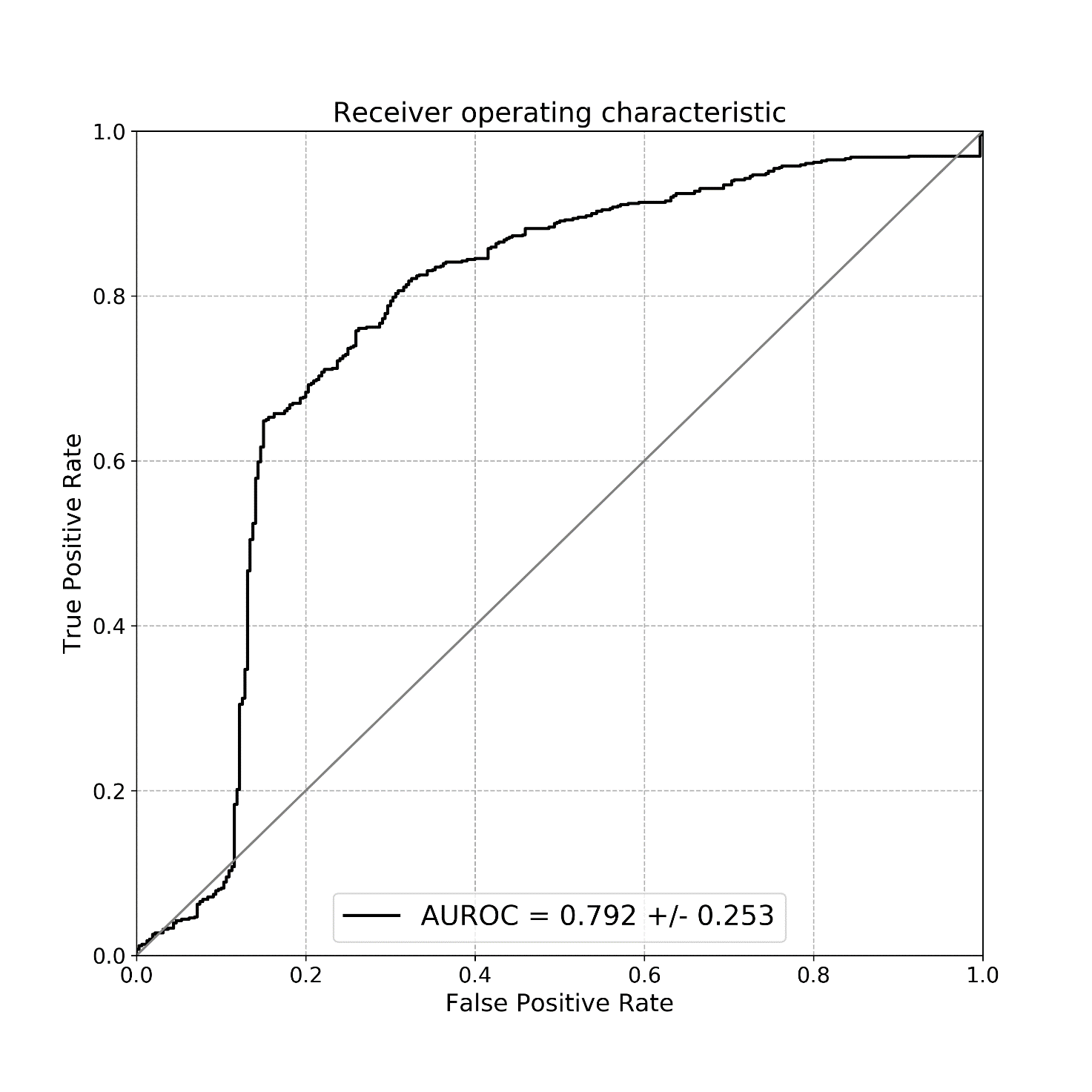


Average area under receiver operating characteristic (AUROC) of regression models built with Reactome pathways, after multiple runs of internal cross-validation to distinguish preclinical systemic sclerosis (PreSSc) patients vs healthy controls patients.
